# Successful Dostarlimab Rechallenge Following Pembrolizumab‐Induced Autoimmune Hemolytic Anemia: A Case Report

**DOI:** 10.1002/ccr3.70776

**Published:** 2025-08-17

**Authors:** Zaid Khamis, Kai Wang, Mohammad Aldalahmeh, Salim Barakat, Nawaraj Adhinkari, Raghad Khassawneh, Bhavya Misri, Dhar Meekoo

**Affiliations:** ^1^ Department of Internal Medicine Northwell Health/Staten Island University Hospital New York New York USA; ^2^ Department of Hematology and Oncology Northwell Health/Staten Island University Hospital New York New York USA; ^3^ Department of Medicine and Surgery Jordan University of Science and Technology Irbid Jordan

**Keywords:** adverse immune events, autoimmune hemolytic anemia, checkpoint inhibitor rechallenge, dostarlimab, immune checkpoint inhibitors, pembrolizumab

## Abstract

Immune checkpoint inhibitors (ICIs) have revolutionized cancer treatment, offering durable responses across multiple malignancies. However, these agents can trigger severe immune‐related adverse events (irAEs), including drug‐induced autoimmune hemolytic anemia (AIHA), which often necessitates treatment discontinuation. While management guidelines for irAEs are well established, the safety and feasibility of ICI rechallenge after resolution of severe hematologic toxicities remain poorly understood. We herein present a case of pembrolizumab‐induced warm autoimmune hemolytic anemia that was successfully rechallenged with dostarlimab. In this case report, we describe a 66‐year‐old female with a history of stage III C2 endometrial cancer who is status post total abdominal hysterectomy, bilateral salpingo‐oophorectomy, and omentectomy. She had completed six cycles of adjuvant chemotherapy with paclitaxel and carboplatin. At her 12‐month follow‐up, elevated CA‐125 levels and imaging (CT Abdomen Pelvis with PET/CT) indicated possible disease recurrence at the vaginal cuff. A subsequent vaginal biopsy confirmed relapse and recurrence of endometrioid adenocarcinoma with squamous differentiation. Given that the tumor is MMR deficient, the patient was started on pembrolizumab along with carboplatin and paclitaxel. However, after the third cycle, she developed IgG‐positive warm autoimmune hemolytic anemia, attributed to pembrolizumab, leading to the discontinuation of the drug. She was treated with steroids, resulting in the resolution of her AIHA, and was then re‐challenged with dostarlimab and is showing promising results thus far. Our case demonstrates that rechallenge with alternative immune checkpoint inhibitors may be feasible in selected patients who have experienced immune‐related adverse events. However, this decision requires careful consideration of multiple factors, including the type and severity of the initial immune‐related adverse event, the potential consequences of recurrence, and the availability of alternative treatment options.

**Summary**

- Rechallenge with dostarlimab may be a safe option for patients who develop pembrolizumab‐induced AIHA, enabling continued immunotherapy in select cases with close monitoring.

## 1 Introduction

Immune checkpoint inhibitors (ICIs) have revolutionized cancer treatment, offering durable responses across multiple malignancies. However, these agents can trigger severe immune‐related adverse events (irAEs), including autoimmune hemolytic anemia (AIHA), which often necessitates treatment discontinuation. While management guidelines for irAEs are well established, the safety and feasibility of ICI rechallenge after resolution of severe hematologic toxicities remain poorly understood. We herein present a case of pembrolizumab‐induced warm autoimmune hemolytic anemia that was successfully rechallenged with dostarlimab.

## 2 Case History/Examination

**FIGURE 1:**
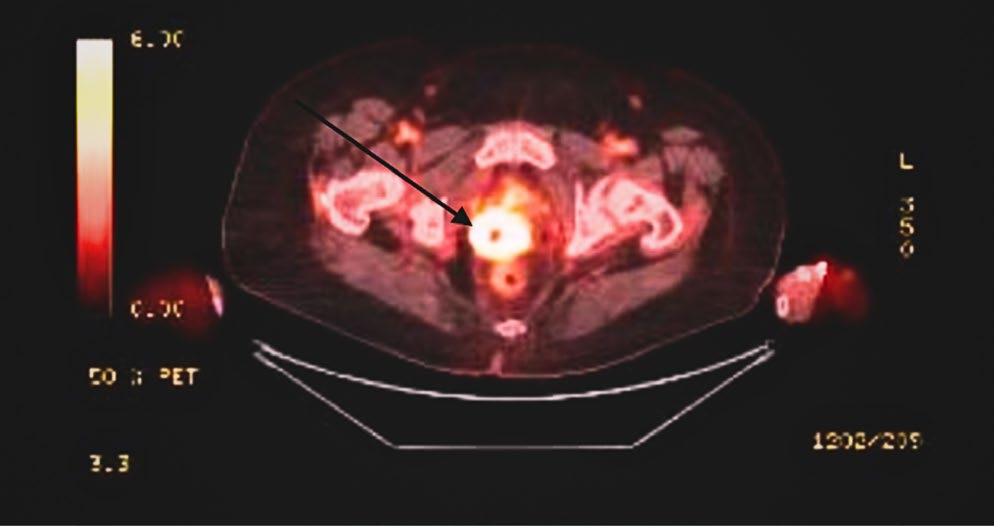
FDG‐PET/CT Imaging of Vaginal Cuff Lesion. The figure displays an axial FDG‐PET/CT image showing an intensely FDG‐avid lesion with a photopenic center, corresponding to the vaginal cuff lesion. The maximum standardized uptake value (SUV) of the lesion is 17.6, indicating significant metabolic activity. The findings suggest biologic tumor activity at this site.

A 66‐year‐old female with a history of stage III C2 endometrial adenocarcinoma who is status post total abdominal hysterectomy with bilateral salpingo‐oophorectomy and omentectomy. After that, she received six cycles of adjuvant chemotherapy with carboplatin and paclitaxel, external beam radiation therapy, and brachytherapy. At her 12‐month follow‐up, elevated CA125 levels prompted further investigation, revealing a vaginal cuff lesion on CT scan. A subsequent PET‐CT showed increased FDG uptake at the vaginal cuff (Figure 1), indicating possible disease recurrence. A vaginal biopsy confirmed adenocarcinoma, endometrioid type, with foci of squamous differentiation and extensive necrosis. The microsatellite status was determined to be MSI‐High. The patient was started on a regimen of carboplatin, paclitaxel, and pembrolizumab. Baseline laboratory tests showed a hemoglobin (Hb) level of 10 g/dL, with normal white blood cell (WBC) and platelet counts, and normal comprehensive metabolic panel (CMP).

## 3 Differential Diagnosis, Investigations, and Treatment

One week after the third cycle of the above regimen, the patient presented with fatigue and poor oral intake, which prompted hospital admission. Laboratory results on admission, summarized in Table 1, showed severe anemia as her Hb fell to 4.9 g/dL, with normal WBC counts and low platelets of 53 × 10^9^/L; MCV was 82 fl, RDW was 14%, and liver enzymes were within normal limits (ALT of 13 U/L and AST of 14 U/L). Differential diagnosis included chemotherapy‐induced bone marrow suppression and hemolytic anemia. Hemolysis panel sent revealed elevated lactate dehydrogenase (LDH) of 420 IU/L, low haptoglobin of < 20 mg/dL, and elevated total bilirubin of 5.2 mg/dL (direct bilirubin levels were not sent); the reticulocyte index was 0.8. A polyspecific direct antiglobulin test, sent 48 h after PRBCs transfusion, came back positive. Further testing with monospecific DAT was positive for IgG, confirming the diagnosis of warm autoimmune hemolytic anemia (AIHA) secondary to pembrolizumab. The patient received blood transfusions and was started on high‐dose oral prednisone (1 mg/kg) and was slowly tapered over 8 weeks. A few days after starting the prednisone, her hemoglobin stabilized around 9 g/dL, with LDH decreasing to 114 IU/L, total bilirubin decreasing to 1.1 mg/dL, and haptoglobin rising to 168 mg/dL.

**TABLE 1:** Significant laboratory findings of our patient.

Items	Day 1^a^	Day 3^a^	6 months follow‐up	After 3 cycles of dostarlimab	1 year follow‐up after 8 cycles of dostarlimab
WBC Count (×10^9^/L)	9	8	8.3	6.38	6.2
Hemoglobin (g/dL)	4.9	8.7	7.9	9.1	10.1
Platelets (×10^9^/L)	53	63	171	167	234
Reticulocytes Index	0.8	NA	NA	1.6	0.7
LDH (IU/L)	420	460	112	NA	136
Haptoglobin (mg/dL)	< 20	< 20	NA	241	201
Total Bilirubin (mg/dL)	5.2	2.9	1.1	0.3	0.2
ALT/AST (U/L)	13/14	14/19	14/16	6/11	10/14

*Note:* Laboratory parameters for our patient from day 1 of her hospital admission due to pembrolizumab induced AIHIA through 3 cycles of dostarlimab therapy.

Abbreviations: ALT, Alanine Aminotransferase; AST, Aspartate Aminotransferase; LDH, Lactate Dehydrogenase; NA, Not Available; WBC, White Blood Cell.

^a^ Of hospital admission. Of note, indirect bilirubin levels were unavailable.

Given the MMR‐deficient status of her tumor and the fact that her hemoglobin and hemolysis panels have stabilized, the decision was made to re‐challenge her with dostarlimab every 3 weeks with close monitoring.

## 4 Conclusion and Results

The patient has been tolerating the treatment with dostarlimab well, with stable hemoglobin levels and no further need for blood transfusions thus far. Her latest hemoglobin level 3 cycles after dostarlimab initiation was 9.1 g/dL (Table 1).

A repeat PET scan after 5 rounds of dostarlimab showed no progression of disease; in fact, it revealed a slight decrease in the size of the previously seen mass on the vaginal cuff. Throughout her treatment on dostarlimab, her vaginal bleeding resolved, and she experienced gradual improvement in functional status, accompanied by weight gain and increased appetite. Additionally, imaging done 6 months later revealed no evidence of disease progression.

Our case demonstrates that rechallenge with alternative immune checkpoint inhibitors may be feasible in selected patients who have experienced immune‐related adverse events. However, this decision requires careful consideration of multiple factors, including the type and severity of the initial immune‐related adverse event, the potential consequences of recurrence, and the availability of alternative treatment options.

## 5 Discussion

Immune checkpoint inhibitors (ICIs) have revolutionized cancer treatment; yet their use is frequently complicated by immune‐related adverse events (IRAEs) that can necessitate treatment discontinuation [1]. Despite the growing prevalence of ICI therapy, current literature provides limited guidance on rechallenge decisions with alternative PD‐1 inhibitors [2].

Our report describes a case of successful immune checkpoint inhibitor rechallenge utilizing dostarlimab in a patient with recurrent endometrial adenocarcinoma who previously developed warm autoimmune hemolytic anemia (AIHA) secondary to pembrolizumab therapy. The initial immune‐related autoimmune hemolytic anemia (irAIHA) achieved complete resolution following systemic corticosteroid therapy with high‐dose prednisone (1 mg/kg) administered on a standardized tapering protocol [3]. This prompt intervention aligns with established management principles outlined by Postow et al., who emphasize that early recognition and aggressive management of hematologic irAEs is critical to successful resolution and potential for subsequent therapy [4].

The incidence of immune checkpoint inhibitor (ICI)‐induced autoimmune hemolytic anemia (AIHA) is relatively rare, occurring in only 0.15%–0.25% of patients across large clinical trials [1]. Despite its rarity, management typically necessitates immediate immunotherapy discontinuation—especially for high‐grade toxicities—and high‐dose corticosteroids, potentially compromising cancer treatment outcomes [5]. This creates a complex clinical decision point regarding potential rechallenge with alternative ICI agents.

While the American Society of Clinical Oncology (ASCO) and European Society for Medical Oncology (ESMO) provide general frameworks for ICI rechallenge, their guidelines offer limited specific direction for hematologic toxicities [5, 6].

Dolladille et al. conducted a systematic cross‐sectional investigation examining ICI rechallenge patterns following immune‐related adverse events. Their analysis of 6123 patients with documented irAEs revealed that only 452 cases (7.4%) underwent subsequent ICI rechallenge with assessable outcomes. Subgroup analysis revealed significantly elevated recurrence rates in patients with preceding hepatic, gastrointestinal, and pulmonary toxicities, specifically hepatitis, colitis, and pneumonitis, respectively. The study also demonstrated significant heterogeneity in physician decision‐making, with clinicians showing a higher propensity to reinitiate immunotherapy after endocrine irAEs compared to more severe manifestations such as hepatitis, colitis, and pneumonitis. While hematologic toxicity recurrence rates were not specifically reported in their study due to limited cases, the study noted equal representation of hematological adverse events between the rechallenge and the non‐rechallenge cohorts [7]. Notably, this study highlighted that clinicians are typically more hesitant to rechallenge patients following hematologic irAEs compared to endocrine or dermatologic manifestations, making our case particularly instructive for clinical practice [7].

Re‐challenging with an alternative PD‐1 inhibitor still carries the risk of developing immune‐related adverse events. However, dostarlimab may be less likely to cause autoimmune hemolytic anemia (AIHA) compared to pembrolizumab. Dostarlimab exhibits low immunogenicity, as evidenced by a low incidence of anti‐drug antibodies, and its IgG4 isotype, which confers minimal binding to Fc gamma receptors and complement protein C1q, which helps reduce the risk of antibody‐dependent cellular cytotoxicity and complement‐mediated tissue damage. Additionally, dostarlimab's dosing regimen is optimized to maintain effective therapeutic concentrations while minimizing peak exposures that could trigger immune‐related adverse events. All in all, while both dostarlimab and pembrolizumab are anti–PD‐1 monoclonal antibodies, subtle differences in their binding epitopes and antibody engineering may further contribute to the observed differences in immune‐related toxicity profiles [8].

The above approach with ICI re‐challenge was supported by recent meta‐analysis data by Zhao et al., which suggests that rechallenge with an alternative ICI agent may be associated with a lower risk of irAE recurrence compared to rechallenge with the same agent. Their findings demonstrate that while cross‐reactivity between different ICI agents can occur, strategic selection of alternative agents with distinct pharmacokinetic profiles may reduce recurrence risk [2].

The implementation of prophylactic immunosuppression during immune checkpoint inhibitor rechallenge remains controversial due to insufficient evidence‐based protocols. While prophylactic measures might theoretically mitigate recurrent immune‐related adverse events, they may potentially compromise anti‐tumor immune responses and present their own adverse effect profile [9]. Furthermore, the optimal timing, duration, and choice of prophylactic agents remain undefined. Our case demonstrated successful rechallenge without prophylaxis; though larger studies are needed to establish optimal protocols. As Postow et al. discuss, the balance between sufficient immunosuppression to prevent irAE recurrence and maintaining anti‐tumor immune activity presents a significant clinical challenge that requires individualized assessment [4].

In light of the above, this paucity of data highlights the critical importance of carefully documented cases to guide clinical practice. Our experience suggests that judicious patient selection may facilitate successful rechallenge with alternative PD‐1 inhibitors following irAIHA, particularly when initial toxicity achieves complete resolution with standard corticosteroid therapy. Nevertheless, this decision requires careful consideration of individual risk–benefit ratios when contemplating therapy re‐initiation.

## Author Contributions

**Zaid Khamis:** conceptualization, investigation, methodology, project administration, resources, supervision, writing – original draft, writing – review and editing. **Kai Wang:** data curation, methodology, project administration, software, validation, writing – original draft, writing – review and editing. **Mohammad Aldalahmeh:** conceptualization, formal analysis, project administration, software, supervision, validation, writing – original draft, writing – review and editing. **Salim Barakat:** investigation, methodology, supervision, validation, visualization, writing – review and editing. **Nawaraj Adhinkari:** investigation, methodology, supervision, writing – review and editing. **Raghad Khassawneh:** conceptualization, project administration, supervision, writing – review and editing. **Bhavya Misri:** conceptualization, data curation, formal analysis. **Dhar Meekoo:** conceptualization, project administration, supervision, writing – review and editing.

## Consent

Written informed consent was obtained from the patient to publish this report in accordance with the journal's patient consent policy and will be provided upon request.

## Conflicts of Interest

The authors declare no conflicts of interest.

## Data Availability

Data available on request from the authors.
